# Global Call to Action: maximize the public health impact of intermittent preventive treatment of malaria in pregnancy in sub-Saharan Africa

**DOI:** 10.1186/s12936-015-0728-x

**Published:** 2015-05-18

**Authors:** R Matthew Chico, Stephanie Dellicour, Elaine Roman, Viviana Mangiaterra, Jane Coleman, Clara Menendez, Maud Majeres-Lugand, Jayne Webster, Jenny Hill

**Affiliations:** Department of Disease Control, London School of Tropical Medicine and Hygiene, London, UK; Department of Clinical Sciences, Liverpool School of Tropical Medicine, Pembroke Place, Liverpool, UK; Maternal and Child Health Integrated Program, Jhpiego, Baltimore, MD USA; RMNCH and HSS Technical Advice & Partnerships Department, The Global Fund to Fight AIDS, Tuberculosis and Malaria, Vernier, Geneva Switzerland; ISGlobal, Barcelona Centre for International Health Research (CRESIB), Hospital Clínic-Universitat de Barcelona, Barcelona, Spain; Access & Product Management, Medicine for Malaria Venture, Geneva, Switzerland

**Keywords:** Intermittent preventive treatment, Malaria, Prevention, Pregnant women, Sub-Saharan Africa

## Abstract

Intermittent preventive treatment of malaria in pregnancy is a highly cost-effective intervention which significantly improves maternal and birth outcomes among mothers and their newborns who live in areas of moderate to high malaria transmission. However, coverage in sub-Saharan Africa remains unacceptably low, calling for urgent action to increase uptake dramatically and maximize its public health impact. The ‘Global Call to Action’ outlines priority actions that will pave the way to success in achieving national and international coverage targets. Immediate action is needed from national health institutions in malaria-endemic countries, the donor community, the research community, members of the pharmaceutical industry and private sector, along with technical partners at the global and local levels, to protect pregnant women and their babies from the preventable, adverse effects of malaria in pregnancy.

## Background

Historic progress in malaria control has resulted in decreased malaria-attributable mortality worldwide by 47 % since 2000 [1]. However, progress in the prevention of malaria in pregnancy (MiP) during the same time period has been less remarkable. Close to 32 million pregnancies occur every year in areas of stable malaria transmission where the World Health Organization (WHO) recommends a three-pronged approach to control MiP: the provision of intermittent preventive treatment in pregnancy (IPTp) using sulphadoxine-pyrimethamine (SP), the use of insecticide-treated nets (ITNs), and effective case management of malaria illness and anaemia [2, 3]. IPTp is a highly cost-effective intervention with the potential to reduce maternal morbidity and neonatal mortality [4]. Meta-analyses of women in their first and second pregnancies have shown that IPTp decreases the incidence of low birth weight (LBW) by 27 %, severe maternal anaemia by 40 %, and neonatal mortality by 38 %, although the evidence on neonatal mortality is less conclusive [5]. IPTp with SP remains effective in preventing the adverse consequences of malaria in pregnancy across a wide range of SP resistance levels [6]. The use of IPTp with SP, together with ITNs, remains essential for protecting pregnant women against malaria infection and the adverse birth outcomes associated with MiP in sub-Saharan Africa [7].

Despite being a straightforward intervention that can be delivered to pregnant women under direct observation during routine antenatal care (ANC) visits, IPTp coverage remains unacceptably low. In 2013, average coverage of at least two doses of IPTp among pregnant women in sub-Saharan African countries was 24 %, well below national and international targets, and only marginally higher than a decade earlier when coverage was 14 % [8]. Just six countries in sub-Saharan Africa have reached the 2005 coverage target of 60 % set by the Roll Back Malaria Partnership (RBM); not one country has reached the 2010 RBM target of 80 % coverage [9, 10]. The most recent World Malaria Report stated that 15 million of the 35 million pregnant women in sub-Saharan Africa malaria-endemic countries did not receive a single dose of IPTp in 2013 [1].

In 2012, WHO updated its IPTp policy to recommend that SP be administered as early as possible during the second trimester and at every scheduled ANC visit thereafter, so long as the doses are given at least one month apart [11]. This update was, in part, aimed at increasing the number of SP doses administered; IPTp has the lowest coverage among all interventions delivered to pregnant women through the ANC platform, including the provision of long-lasting insecticide-treated nets [12]. The discrepancy between high ANC attendance (75 % of women in sub-Saharan Africa attend at least twice) and low IPTp coverage points to substantial missed opportunities at ANC facilities [8, 13].

### What can be done to close this gap?

In 2013, the RBM MiP Working Group released a Consensus Statement, calling on stakeholders to renew their commitment to scale-up malaria control interventions that target pregnant women and to take action that would: (1) harmonize national reproductive health and malaria policies in support of WHO’s three-pronged strategy against MiP; (2) strengthen health systems; and (3) improve monitoring and evaluation of MiP programme effectiveness. The recent RBM report on the contribution of malaria control to maternal and newborn health highlighted key programme areas for improvement that will contribute to the scale-up of MiP interventions [14]. The report states that many barriers to IPTp uptake are common across countries and can be overcome relatively easily and rapidly [14]. These obstacles include a lack of integration and coordination between national reproductive health and malaria control programmes. Better coordination is central to preventing MiP given that interventions are delivered through ANC clinics with management from staff in reproductive health services and technical oversight from counterparts in malaria control. Other obstacles include: inconsistent, unclear and poorly disseminated information on IPTp policy; confusion among healthcare providers as to when and how to administer IPTp; SP stock-outs at ANC clinics [15], and well-documented barriers to accessing ANC services [16]. Additional obstacles relate to broader health systems issues and necessitate strengthening the health system generally and ANC services specifically.

In order to reinforce stakeholder commitment to increasing IPTp coverage, a ‘Global Call to Action for the scale-up of IPTp’ was held during the 63rd Annual Meeting of the American Society of Tropical Medicine and Hygiene [17]. Based on previous reports, a set of recommendations was developed and further elaborated through a series of consultations among key stakeholders [14, 18]. The recommendations address key challenges to the scale-up of IPTp that are required to maximize its public health impact. The ongoing revision of the WHO ANC guidelines represents a strategically crucial opportunity for MiP to be re-prioritized as a core component of focused ANC.

### Recommendations by key stakeholders

#### National level

Commitment from malaria-endemic countries to scale-up IPTp is pivotal to creating momentum that is sustained—if not accelerated—towards reaching coverage targets. Immediate actions as listed in Table 1 can be pursued without delay and, indeed, are critical to curbing the adverse consequences of MiP.

**Table 1:** Recommended actions for the scale-up of IPTp

**Health system building blocks**	**Key health-system programme areas for the scale-up of IPTp** [15]	**Who**	**What**
**Governance**	**Integration** between programmes at the ANC platform	➢ National health institutions in malaria-endemic countries	➢ Establish or strengthen national technical working groups on, or including, MiP to improve the quality of service delivery of focused ANC in both the public and private sector^a^
	**Policy** ensuring consistent, clear and widely disseminated information	➢ National health institutions in malaria-endemic countries	➢ Disseminate clear guidelines with simplified language for IPTp based on the WHO 2012 policy update and ensure that IPTp is in national strategic and operational plans
**Financing**	**Finance** for health systems strengthening with focus on the ANC platform and removing costs as a barrier to ANC and IPTp uptake as well as support operational research	➢ National health institutions in malaria-endemic countries	➢ Reduce, or eliminate where possible, ANC fees to overcome cost as a barrier to ANC access for end-users and the uptake of IPTp
		➢ Donor community	➢ Increase levels of financial support for health systems strengthening with focus on the ANC platform
			➢ Provide support for operational research to improve the quality of service delivery and increase IPTp coverage
			➢ Promote the inclusion of IPTp and malaria control in pregnancy within grant proposals (e.g. The Global Fund and President’s Malaria Initiative) from endemic countries, alongside all programme areas of health systems strengthening
			➢ Support private sector engagement in increasing funding and support for improved IPTp coverage
**Human Resource**	**Capacity development** to improve healthcare provider knowledge and skills for the delivery of IPTp	➢ National health institutions in malaria-endemic countries	➢ Ensure healthcare providers in the public and private sectors have access to and training on the updated IPTp guidelines
**Product & Technology**	**Commodities** supply chain management strengthening	➢ National health institutions in malaria-endemic countries	➢ Earmark funding for procurement for IPTp delivery through ANC channels in both the public and private sector to prevent stock-outs
		➢ Pharmaceutical industry	➢ Meet the demand for SP procurement and register quality SP in all malaria-endemic countries
			➢ Meet the demand for procurement and register low-dose folic acid in all malaria-endemic countries
			➢ Identify and develop new drugs suitable for use as IPTp
**Health Management Information Systems**	**Monitoring and evaluation** to assess IPTp effectiveness and progress and challenges to delivery and uptake at national and local levels	➢ National health institutions in malaria-endemic countries	➢ Ensure IPTp coverage indicators are in line with new policy
			➢ Use District Health Management Information System (HMIS) to measure coverage of IPTp, report changes in coverage, identify bottlenecks and take necessary action
		➢ Research community; National health institutions in malaria-endemic countries and WHO	➢ Collaborate with WHO and Ministries of Health to establish monitoring and evaluation plans for:
			◦ SP resistance and its impact on IPTp-SP effectiveness;
			◦ Reducing malaria transmission and its impact on IPTp-SP effectiveness;
			◦ Data on uptake and coverage of IPTp from district and facility levels that will help to identify implementation bottlenecks.
**Service Delivery**	**Quality assurance** to monitor implementation and eliminate gaps in service delivery	➢ National health institutions in malaria-endemic countries	➢ Explore innovative opportunities in the community for service delivery, both to extend antenatal clinic-based programmes and to serve women where ANC services are under-developed
		➢ Research community	➢ Evaluate alternative strategies for the delivery of IPTp in hard-to-reach populations or communities
	**Community engagement** to promote ANC, raise awareness of IPTp benefits and increased uptake of IPTp	➢ National health institutions in malaria-endemic countries	➢ Undertake targeted communication campaigns at both the community and facility levels to promote ANC attendance among pregnant women and to raise awareness of the benefits of IPTp
		➢ Research community	➢ Identify, promote and evaluate successful behaviour change communication strategies based on the RBM toolkit^b^ to:
			◦ Increase demand and acceptance of IPTp-SP by pregnant women;
			◦ Improve healthcare provider attitudes and performance;

Abbreviations: *ANC* antenatal care, *IPTp* intermittent preventive treatment for malaria in pregnancy, *RBM* Roll Back Malaria Partnership, *SP* sulphadoxine-pyrimethamine, *WHO* World Health Organization

^a^Non-governmental sector in this context includes private practitioners, faith-based organizations and other non-governmental organizations that provide health services outside of the Ministry of Health

^b^ http://www.rollbackmalaria.org/files/files/partnership/wg/wg_communication/docs/Malaria-BCC-Indicators-Reference-Guide.pdf

On the supply side, endorsement of IPTp and deliberate integration of ANC services is needed across public health programmes including reproductive health, malaria, HIV and TB. This will require that healthcare providers are trained on, and have access to, the updated WHO guidelines on IPTp administration. A recent review of national MiP policies, guidelines, and training tools in five countries found that many documents were outdated and/or discordant between national malaria programmes and reproductive health programmes, which could lead to incorrect practices (such as withholding IPTp due to misunderstanding the appropriate gestational age for administering IPTp) among ANC healthcare providers [19]. Low-cost visual job aids for healthcare providers that communicate clear messages on the timing, spacing and dosage of IPTp as described elsewhere [20] should be widely used. Previous experience has shown that community-based promotion or distribution of IPTp, alongside promotion of ANC services, is effective at increasing coverage in some settings [21–24]. Earmarked funding that ensures a steady supply of quality SP and low-dose folic acid (0.4 mg) is recommended for use in conjunction with IPTp-SP (a daily dose of folic acid that is equal or greater than 5 mg has been shown to counteract the anti-malarial effect of SP [25, 26]), and that these are to be provided free-of-charge to pregnant women at ANC clinics. Simply making sure that drinking water and cups are available for women to use is key to preventing countless missed opportunities.

On the demand side, broad community engagement, targeted promotional campaigns and empowering women with information on the benefits of IPTp and other services provided at ANC facilities are also important. A systematic review identified several barriers to IPTp uptake including a lack of community knowledge about the advantages of IPTp, the safety of SP use in pregnancy, the recommended dosing schedule for IPTp, and whether SP can be taken on an empty stomach [15]. Addressing the main barriers to ANC attendance such as out-of-pocket costs, negative staff attitudes often expressed verbally and non-verbally towards patients, and long waiting times could contribute to better uptake of IPTp as well as other ANC services across socio-economic groups and reduce inequities to access [16].

The non-governmental sector—comprised of private practitioners, faith-based organizations and other non-governmental organizations—provides up to 60 % of health services in some countries [27]. Consequently, this sector is critical to the scale-up of IPTp and must be incorporated into efforts aimed at health system strengthening including health worker training, supportive supervision, and preventing SP stock outs. Continuous monitoring and evaluation through the use of health management information systems will enable countries to track their own progress and identify bottlenecks in the delivery of IPTp. This will require updating information systems and ANC registers to collect data on IPTp doses given at every ANC visit, in line with the updated WHO policy recommendation. Civil society can also play an important role by reinforcing the importance of IPTp to communities at risk of malaria and holding governments accountable for the delivery of IPTp.

### International level

At the international level, the RBM MiP Working Group provides strategic advice and technical guidance on best practices for the scale-up of MiP interventions, including IPTp. Donors and multilateral organizations such as the Global Fund, the World Bank, the Bill and Melinda Gates Foundation, UNICEF and international aid agencies [*e.g.* the President’s Malaria Initiative (US Agency for International Development and US Centers for Disease Control and Prevention) and the UK Department for International Development], should work in close partnership with the RBM MiP working group to advocate for and to support health systems strengthening and operational research that is focused on IPTp, both of which are required to meet targets and sustain results.

The pharmaceutical industry has a fundamental role in producing good-quality, safe, well-tolerated, efficacious, and affordable medicines that are accessible to populations in need of treatment. To this end, the pharmaceutical industry should consider working with the 36 malaria-endemic countries that have an IPTp policy to facilitate the availability of SP and low-dose folic acid [25, 26]. Procurement of SP has been a challenge due to its transition from the first-line treatment for uncomplicated malaria to comparatively limited use in IPTp, and more recently, seasonal chemoprophylaxis in children when combined with amodiaquine. Together with the research community, the pharmaceutical industry is encouraged to play an important role in identifying alternative drugs that can be used for IPTp in areas where SP may be a sub-optimal therapy due to increasing parasite resistance.

The research community needs to facilitate implementation of evidence-based practices, while continuing to play a key role in supporting the scale-up of IPTp. Operational research is needed to evaluate strategies—both existing and new—that are designed to increase antenatal care-seeking behaviour, especially early in pregnancy, and to improve healthcare provider attitudes exhibited towards pregnant women who present at ANC facilities. Further operational research is needed to evaluate community distribution of IPTp that seeks to increase IPTp uptake while not discouraging ANC attendance. Continued collaboration with WHO and Ministries of Health will be required to monitor IPTp effectiveness and to foster data-driven decision-making by programme managers to identify bottlenecks in IPTp provision and prioritize appropriate action.

Prevention of MiP is a core component of maternal and newborn health. IPTp is a highly cost-effective tool that has been proven to prevent maternal and neonatal deaths and should be made a top priority. International coverage targets can be reached through a combination of powerful advocacy, firm political and financial commitments, and strong local, national and international partnerships. An immediate need is for countries to develop consensus around country- and district-level action plans for the scale-up of IPTp that have been adapted to local requirements with support from technical partners.

## Abbreviations

ANC: Antenatal care
HMIS: Health management information systems
IPTp: Intermittent preventive treatment of malaria in pregnancy
ITNs: Insecticide treated nets
MiP: Malaria in Pregnancy
RBM: The Roll Back Malaria Partnership
SP: Sulphadoxine-pyrimethamine
WHO: World Health Organization
